# Antiobesity, antidiabetic, and antioxidant activities of selected *Rubia* species (Rubiaceae) and LC–MS/MS-based chemical profiling

**DOI:** 10.55730/1300-0144.6374

**Published:** 2026-04-01

**Authors:** Semih BULUT, Ahmet KAHRAMAN, Ayşenur IŞIK, Mustafa Abdullah YILMAZ

**Affiliations:** 1 Department of Pharmacognosy, Faculty of Pharmacy, Süleyman Demirel University, Isparta, Turkiye; 2 Department of Molecular Biology and Genetics, Faculty of Engineering and Natural Sciences, Uşak University, Uşak, Turkiye; 3 Faculty of Pharmacy, Süleyman Demirel University, Isparta, Turkiye; 4 Department of Analytical Chemistry, Faculty of Pharmacy, Dicle University, Diyarbakır, Turkiye

**Keywords:** Diabetes mellitus, antioxidants, enzyme inhibitors, *Rubia* species

## Abstract

**Background/aim:**

*Rubia* species (Rubiaceae) are recognized as medicinal and industrial plants exhibiting diverse biological activities. This study was conducted to evaluate the antioxidant, antidiabetic, and antiobesity potential of extracts prepared from *Rubia davisiana* Ehrend. (an endemic species), *R. peregrina* L., and *R. rotundifolia* Banks & Sol., which grow naturally in Türkiye.

**Materials and methods:**

The antioxidant activity of 80% ethanol extracts prepared from the aerial parts was evaluated using 2,2’-azino-bis(3-ethylbenzothiazoline-6-sulfonate) (ABTS) and 2,2-diphenyl-1-picrylhydrazyl (DPPH) radical scavenging assays, ferric reducing power, metal chelating capacity, and total antioxidant capacity assays. Inhibition assays for α-glucosidase and α-amylase were performed to evaluate antidiabetic activity, whereas a pancreatic lipase inhibition assay was conducted to assess antiobesity activity. Additionally, the quantitative chemical composition of the extracts was determined using liquid chromatography–tandem mass spectrometry (LC–MS/MS) analysis.

**Results:**

The total phenolic content of the extracts ranged from 52.15 to 66.77 mg GAE/g extract. All extracts exhibited strong DPPH radical scavenging activity at concentrations of 1 and 2 mg/mL. ABTS radical scavenging activity and ferric reducing power increased in a concentration-dependent manner. Among the tested extracts, *R. rotundifolia* exhibited the highest total antioxidant capacity (147.35 mg ascorbic acid equivalent [AAE]/g extract). *R. rotundifolia* extract demonstrated stronger α-amylase inhibitory activity than the other extracts at all tested concentrations. At a concentration of 2 mg/mL, *R. rotundifolia* extract exhibited 74.70% inhibition of α-glucosidase activity. LC–MS/MS analysis revealed that the aerial parts of *R. rotundifolia* were rich in quinic acid and chlorogenic acid.

**Conclusion:**

The antidiabetic and antiobesity activities of extracts obtained from all studied species were evaluated for the first time. In conclusion, the α-glucosidase inhibitory and antioxidant activities of the extract prepared from the aerial parts of *R. rotundifolia* were more pronounced. Further studies are warranted to investigate fractionation processes and bioactivity-guided isolation of active constituents.

## 1. Introduction

Diabetes mellitus, characterized by chronic hyperglycemia, is among the diseases with rapidly increasing global prevalence. It has been estimated that diabetes mellitus will affect approximately 693 million adults worldwide by 2045. Complications of diabetes lead to increased mortality and reduced quality of life [1]. Oxidative stress has been identified as a major contributor to the development of diabetic complications. Increased free radical production and impairment of antioxidant defense mechanisms may contribute to the development of diabetes mellitus [2]. Controlling postprandial blood glucose elevation resulting from carbohydrate hydrolysis represents a key therapeutic strategy in the management of diabetes mellitus [3]. The enzymes α-amylase and α-glucosidase are responsible for carbohydrate hydrolysis, and inhibition of these enzymes can stabilize postprandial blood glucose levels. Therefore, they are frequently targeted in studies evaluating antidiabetic activity [4,5].

The increasing prevalence of type 2 diabetes mellitus has been strongly associated with obesity, and approximately 90% of individuals with type 2 diabetes are overweight. The shared pathophysiological mechanisms of obesity and diabetes are largely attributed to insulin deficiency and insulin resistance [6]. By 2030, a substantial proportion of the global population is projected to become obese due to the rising prevalence of obesity [7]. Inhibition of fat digestion or prevention of lipid absorption into the bloodstream represents an important therapeutic approach in the management of diabetes mellitus and obesity [8]. Accordingly, inhibition of pancreatic lipase, a key enzyme involved in lipid digestion and absorption, is considered a major therapeutic target [9,10].

Rubiaceae is one of the largest plant families, comprising approximately 650 genera and 13,000 species worldwide [11]. Species of the genus *Rubia* (Rubiaceae) grow naturally across diverse geographical regions. Several *Rubia* species, including *Rubia cordifolia* L., *Rubia akane* Nakai, *Rubia tinctorum* L., and *Rubia peregrina* L., have been recognized for their pharmacological and medicinal value [12]. These species have traditionally been used in the management of rheumatism, wound healing, and inflammatory conditions. In Türkiye, five *Rubia* species—*R. davisiana* Ehrend. (an endemic species restricted to Antalya), *R. peregrina*, *R. rotundifolia* Banks & Sol., *R. tinctorum*, and *R. tenuifolia* d’Urv.—are naturally distributed [13]. Limited research has been conducted on these species, with the exception of *R. tinctorum* [14–16].

The effects of *R. davisiana*, *R. peregrina*, and *R. rotundifolia* on α-glucosidase, α-amylase, and pancreatic lipase have not yet been investigated. Accordingly, this study aimed to evaluate the antioxidant, antidiabetic, and antiobesity activities of these species. Total flavonoid and total phenolic contents of the extracts were also determined using spectrophotometric methods.

## 2. Materials and methods

### 2.1. Plant material

The *Rubia* species used in this study were collected and identified by Prof. Dr. Ahmet Kahraman (a coauthor of this study). Voucher specimens are deposited in the GUL Herbarium, Süleyman Demirel University, Isparta, Türkiye, and detailed collection information is presented in Table 1.

### 2.2. Preparation of extracts

The aerial parts of the plant materials (10 g) were extracted with 200 mL of 80% ethanol for 16 h. The extracts were subsequently filtered and concentrated under reduced pressure using a rotary evaporator at 40 °C. All procedures were repeated three times, and the resulting crude extracts were pooled.

### 2.3. Determination of total phenolic content

The total phenolic content of the extracts (1 mg/mL) was determined using the Folin–Ciocalteu method [17]. An aliquot of the extract (20 μL) was mixed with 80% ethanol, followed by the addition of 10% Folin–Ciocalteu reagent. Subsequently, 80 μL of 7.5% sodium carbonate solution was added to the reaction mixture, and the mixture was incubated for 30 min at room temperature. The absorbance was measured at 760 nm using a spectrophotometer. Aliquots (20 μL) of gallic acid standard solutions (0.01, 0.05, 0.25, 0.5, and 1 mg/mL) were used to construct a calibration curve.

### 2.4. Determination of total flavonoid content

Sodium acetate (1 M, CH_3_COONa) and 10% AlCl_3_ solutions were prepared for the determination of total flavonoid content. Subsequently, 95% ethanol, 20 μL of CH_3_COONa solution, and 20 μL of AlCl_3_ solution were added to 100 μL of the extract (1 mg/mL), and the final volume was adjusted to 1 mL with distilled water. The reaction mixture was incubated for 30 min at room temperature. The absorbance of the extracts was measured at 415 nm. Aliquots (20 μL) of quercetin standard solutions (0.01, 0.05, 0.25, 0.5, and 1 mg/mL) were used to construct a calibration curve [18].

### 2.5. Antioxidant activity assays

#### 2.5.1. DPPH radical scavenging activity

A 1 mM 2,2-diphenyl-1-picrylhydrazyl (DPPH) solution was prepared to evaluate radical scavenging activity. Aliquots (80 μL) of the extract or reference standard (ascorbic acid) were transferred into a microplate well, and 1 mM DPPH solution was added. After 30 min of incubation in the dark, absorbance was measured at 520 nm. In this assay, the extracts and the reference standard were tested at concentrations of 0.5, 1, and 2 mg/mL. The same procedure was applied to the reference standard (80 μL) [19]. Radical scavenging activity (%) was calculated using the following equation:


$$\text{Radical scavenging activity }(\%)=[({\text{A}}_{\text{C}}-{\text{A}}_{\text{S}})/{\text{A}}_{\text{C}}]\times 100$$

In this equation, A represents absorbance, C represents the control reaction, and S represents the test sample.

#### 2.5.2. Metal chelating activity

The extracts and the reference standard were prepared at concentrations of 0.5, 1, and 2 mg/mL. Aliquots (100 μL) of the extracts and reference standard were transferred into the wells of a 96-well microplate. Subsequently, 20 μL of 5 mM ferrozine solution and 10 μL of 2 mM FeCl_2_ solution were added to each well. After 10 min of incubation at room temperature, absorbance was measured at 562 nm [20]. Metal chelating activity (%) was calculated using the following equation:


$$\text{Metal chelating activity }(\%)=[({\text{A}}_{\text{C}}-{\text{A}}_{\text{S}})/{\text{A}}_{\text{C}}]\times 100$$

In this equation, A represents absorbance, C represents the control reaction, and S represents the test sample.

#### 2.5.3. Ferric reducing power

The extracts and the reference standard (quercetin) were prepared at concentrations of 0.5, 1, and 2 mg/mL. Aliquots (50 μL) of the extracts were mixed with 1% (w/v) potassium ferricyanide [K_3_Fe(CN)_6_] solution and 50 μL of phosphate buffer (pH 7.2). The reaction mixture was incubated at 37 °C for 60 min. Subsequently, 10% trichloroacetic acid (C_2_HCl_3_O_2_) solution was added to the mixture, and absorbance was measured at 700 nm. Then, 50 μL of 0.1% iron(III) chloride solution was added to the mixture, and a second absorbance measurement was performed. The same procedure was applied to the reference standard (50 μL) [21]. Ferric reducing power was expressed as the difference between the absorbance measured after the addition of iron(III) chloride and the initial absorbance.

#### 2.5.4. Total antioxidant capacity

Ammonium molybdate, sulfuric acid, and sodium phosphate monobasic were mixed, and the total volume was adjusted to 10 mL with distilled water to obtain the molybdate reagent. The prepared reagent was added to 100 μL of the *Rubia* extract (1 mg/mL), and the reaction mixture was incubated at 90 °C for 90 min. The absorbance was then measured at 695 nm. Ascorbic acid standard solutions (0.01, 0.05, 0.25, 0.5, and 1 mg/mL) were prepared to construct a calibration curve [22].

#### 2.5.5. ABTS radical scavenging activity

The extracts and the reference standard were prepared at concentrations of 0.5, 1, and 2 mg/mL. A 7 mM solution of 2,2’-azino-bis(3-ethylbenzothiazoline-6-sulfonate) (ABTS) was prepared by dissolving the compound in distilled water. Subsequently, 2.45 mM potassium peroxodisulfate was mixed with the 7 mM ABTS solution. The mixture (7.5 mL) was incubated at 20 °C for 16 h in the dark, after which phosphate buffer (pH 7.4) was added. Aliquots (10 μL) of the extracts were mixed with the ABTS working solution, and after 6 min of incubation, absorbance was measured at 734 nm. Gallic acid was used as the reference standard in this assay. The same procedure was applied to the reference standard (10 μL) [23]. ABTS radical scavenging activity (%) was calculated using the following equation:


$$\text{ABTS radical scavenging activity }(\%)=[({\text{A}}_{\text{C}}-{\text{A}}_{\text{S}})/{\text{A}}_{\text{C}}]\times 100$$

In this equation, A represents absorbance, C represents the control reaction, and S represents the test sample.

### 2.6. Enzyme inhibition assays

#### 2.6.1. α-glucosidase inhibition assay

The extracts and the reference standard (acarbose) were prepared at concentrations of 0.5, 1, and 2 mg/mL. Aliquots (10 μL) of the extract were added to the wells of a 96-well microplate, followed by 60 μL of phosphate buffer (pH 6.5) and α-glucosidase (Type IV, 3 U/mL). The reaction mixture was incubated at 37 °C for 15 min, after which 10 μL of 20 mM 4-nitrophenyl α-D-glucopyranoside solution was added to each well. Following incubation at 37 °C for 35 min, absorbance was measured at 405 nm. The same procedure was applied to the reference standard (10 μL) [24].

#### 2.6.2. α-amylase inhibition assay

The extracts and the reference standard (acarbose) were prepared at concentrations of 0.5, 1, and 2 mg/mL. Aliquots (15 μL) of the extract were added to the wells of a 96-well microplate, followed by α-amylase (4 U/mL), 15 μL of phosphate buffer (pH 6.9), and 15 μL of 0.5% (w/v) potato starch solution. The reaction mixture was incubated at 37 °C for 15 min. Subsequently, 30 μL of 3,5-dinitrosalicylic acid (DNS) reagent containing potassium sodium tartrate (5.31 M) and DNS (96 mM) was added. The reaction mixture was heated at 80 °C for 40 min to terminate the enzymatic reaction. Absorbance was measured at 540 nm. The same procedure was applied to the reference standard (15 μL) [25].

#### 2.6.3. Pancreatic lipase inhibition assay

The extracts and the reference standard (orlistat) were prepared at concentrations of 0.5, 1, and 2 mg/mL. A 1 mM EDTA solution and a 10 mM 4-morpholinepropanesulfonic acid (MOPS) solution were mixed to prepare 18 mL of MOPS buffer (pH 6.8). Pancreatic lipase (1 mg/mL) from porcine pancreas was dissolved in MOPS buffer and gently vortexed to ensure complete homogenization. Subsequently, 50 mL of Tris buffer (pH 7.4) containing 5 mM calcium chloride and 100 mM Tris-HCl was prepared. Aliquots of the extract (20 μL), pancreatic lipase (15 μL), and Tris buffer (150 μL) were added to the wells of a 96-well microplate. The reaction mixture was incubated at 37 °C for 15 min, after which 15 μL of 5 mM 4-nitrophenyl butyrate solution was added to each well. Following incubation at 37 °C for 30 min, absorbance was measured at 405 nm. The same procedure was applied to the reference standard (20 μL) [26].

### 2.7. Determination of chemical composition by LC–MS/MS

The analysis was performed according to the method described by Yilmaz [27]. A total of 53 phytochemicals were analyzed using ultra-high-performance liquid chromatography (Shimadzu, Kyoto, Japan) coupled with tandem mass spectrometry (MS/MS). A reversed-phase column (Agilent Poroshell 120 EC-C18, 2.7 μm, 150 mm × 2.1 mm; Agilent Technologies, Santa Clara, CA, USA) was used for chromatographic separation. The mobile phase consisted of solvent A (H_2_O + 5 mM NH_4_HCO_2_ + 0.1% CH_2_O_2_) and solvent B (CH_3_OH + 5 mM NH_4_HCO_2_ + 0.1% CH_2_O_2_). The gradient program was as follows: 20%–100% B (0–25 min), 100% B (25–35 min), and 20% B (35–45 min). The injection volume was 5 μL, the flow rate was 0.5 mL/min, and the column temperature was maintained at 40 °C. A Shimadzu LCMS-8040 triple quadrupole mass spectrometer (Shimadzu, Kyoto, Japan) equipped with an electrospray ionization source was used for detection. The MS operating conditions were as follows: desolvation line temperature, 250 °C; nebulizing gas (N_2_) flow rate, 3 L/min; drying gas (N_2_) flow rate, 15 L/min; interface temperature, 350 °C; and heat block temperature, 400 °C.

### 2.8. Statistical analysis

All analyses were performed in triplicate. All results were expressed as mean ± standard deviation (SD). Statistical analyses were performed using GraphPad InStat (GraphPad Software, San Diego, CA, USA) and Microsoft Excel (Microsoft Corp., Redmond, WA, USA). Differences were considered statistically significant at p < 0.05.

## 3. Results

The total phenolic contents of all extracts ranged from 52.15 ± 3.00 to 66.77 ± 1.24 mg gallic acid equivalents (GAE)/g extract. The total flavonoid contents ranged from 48.08 ± 2.16 to 59.44 ± 1.52 mg quercetin equivalents (QE)/g extract. The highest total phenolic and flavonoid contents were observed in *R. davisiana* extract. All extracts exhibited higher total phenolic contents than total flavonoid contents. Despite its high phenolic and flavonoid contents, *R. davisiana* extract did not exhibit strong inhibitory activity against the tested enzymes. The yields, total flavonoid contents, and total phenolic contents of the *Rubia* extracts are presented in Table 2.

All extracts exhibited both DPPH and ABTS radical scavenging activities; however, the DPPH radical scavenging activity was more pronounced. In the ABTS radical scavenging assay, the highest activity was observed in *R. rotundifolia* extract (33.94 ± 0.93%). At a concentration of 2 mg/mL, the DPPH radical scavenging activities of all extracts were comparable to that of ascorbic acid (90.17 ± 0.14%), which was used as the reference standard. The tested extracts exhibited metal chelating activity only at 2 mg/mL. The highest metal chelating activity was observed in *R. rotundifolia* extract (21.16 ± 1.23%), whereas EDTA, used as the reference standard, exhibited markedly higher activity (99.95 ± 0.09%) at the same concentration. In the ferric reducing power assay, *R. davisiana* and *R. rotundifolia* extracts demonstrated moderate reducing activity at 2 mg/mL (absorbance values of 1.820 ± 0.13 and 1.140 ± 0.10, respectively). The absorbance values increased in a concentration-dependent manner. The antioxidant activity results of the extracts are presented in Table 3. Furthermore, *R. rotundifolia* extract exhibited the highest total antioxidant capacity (147.35 ± 7.54 mg (AAE)/g extract).

The inhibitory effects of the extracts on α-amylase, α-glucosidase, and pancreatic lipase were evaluated. None of the extracts prepared from *R. davisiana* exhibited inhibitory activity against α-glucosidase. At 2 mg/mL, *R. peregrina* extract exhibited moderate α-glucosidase inhibitory activity (35.95 ± 1.77%), whereas no significant activity was observed at lower concentrations. The α-glucosidase inhibitory activity of *R. rotundifolia* extract increased in a concentration-dependent manner. At 2 mg/mL, *R. rotundifolia* extract exhibited the highest α-glucosidase inhibitory activity (74.70 ± 0.63%); however, this activity was lower than that of acarbose (99.95 ± 0.38%). Compared with acarbose (93.20 ± 1.28%), the extracts exhibited either no inhibitory activity or only weak activity against α-amylase. Among the tested samples, only *R. rotundifolia* extract inhibited α-amylase at all investigated concentrations and demonstrated higher activity compared with the other plant extracts. At 2 mg/mL, *R. peregrina* and *R. rotundifolia* extracts exhibited weak pancreatic lipase inhibitory activity (12.65 ± 2.91% and 13.18 ± 0.64%, respectively). Although *R. rotundifolia* extract exhibited the highest pancreatic lipase inhibitory activity, this activity was considerably lower than that of orlistat (63.85 ± 1.63%), which was used as the reference drug. The enzyme inhibition assay results are presented in Table 4.

**Figure 1:**
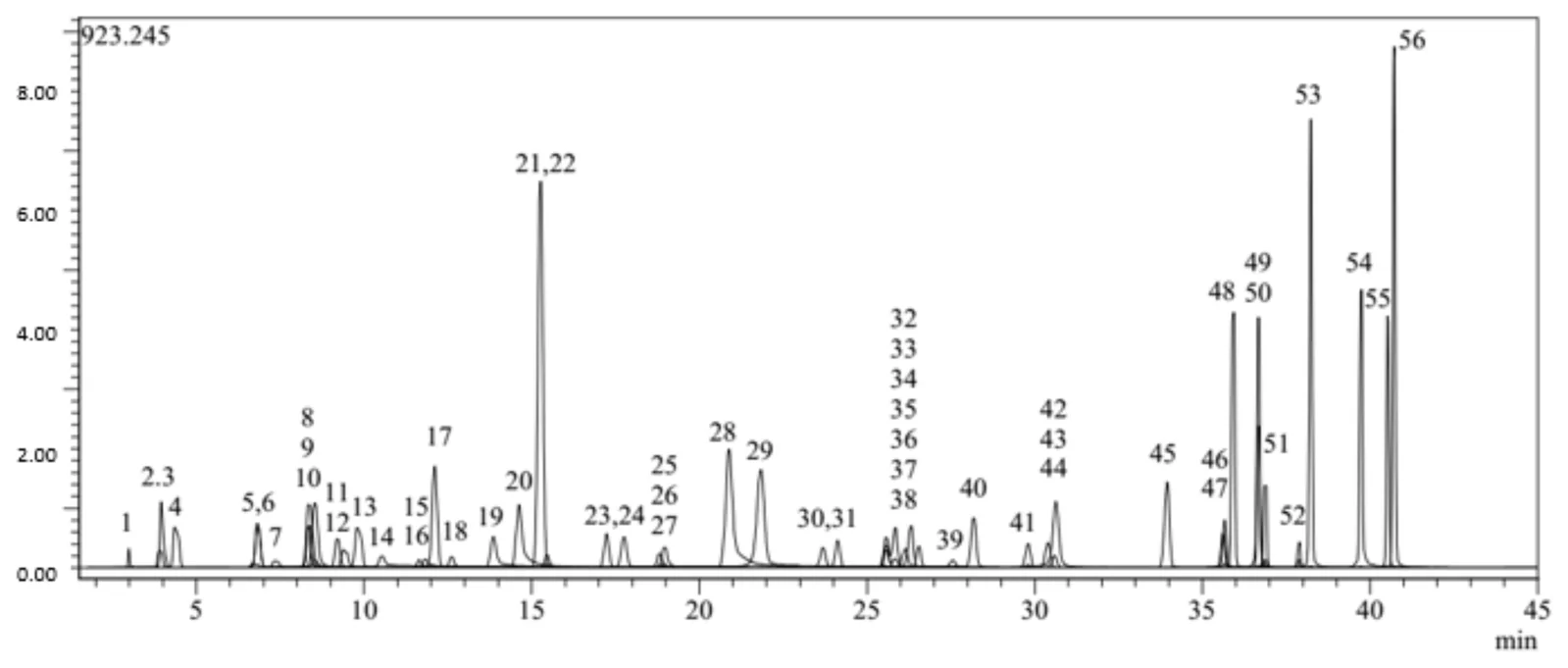
LC–MS/MS chromatogram of the standard compounds. 1. Quinic acid, 2. Fumaric acid, 3. Aconitic acid, 4. Gallic acid, 5. Epigallocatechin, 6. Protocatechuic acid, 7. Catechin, 8. Gentisic acid, 9. Chlorogenic acid, 10. Protocatechuic aldehyde, 11. Tannic acid, 12. Epigallocatechin gallate, 13. Cynarin, 14. 4-hydroxybenzoic acid, 15. Epicatechin, 16. Vanillic acid, 17. Caffeic acid, 18. Syringic acid, 19. Vanillin, 20. Syringic aldehyde, 21. Daidzin, 22. Epicatechin gallate, 23. Piceid, 24. p-Coumaric acid, 25. Ferulic acid-D3-IS, 26. Ferulic acid, 27. Sinapic acid, 28. Coumarin, 29. Salicylic acid, 30. Cyanoside, 31. Miquelianin, 32. Rutin-D3-IS, 33. Rutin, 34. Isoquercitrin, 35. Hesperidin, 36. o-Coumaric acid, 37. Genistin, 38. Rosmarinic acid, 39. Ellagic acid, 40. Cosmosiin, 41. Quercitrin, 42. Astragalin, 43. Nicotiflorin, 44. Fisetin, 45. Daidzein, 46. Quercetin-D3-IS, 47. Quercetin, 48. Naringenin, 49. Hesperetin, 50. Luteolin, 51. Genistein, 52. Kaempferol, 53. Apigenin, 54. Amentoflavone, 55. Chrysin, 56. Acacetin.

**Figure 2:**
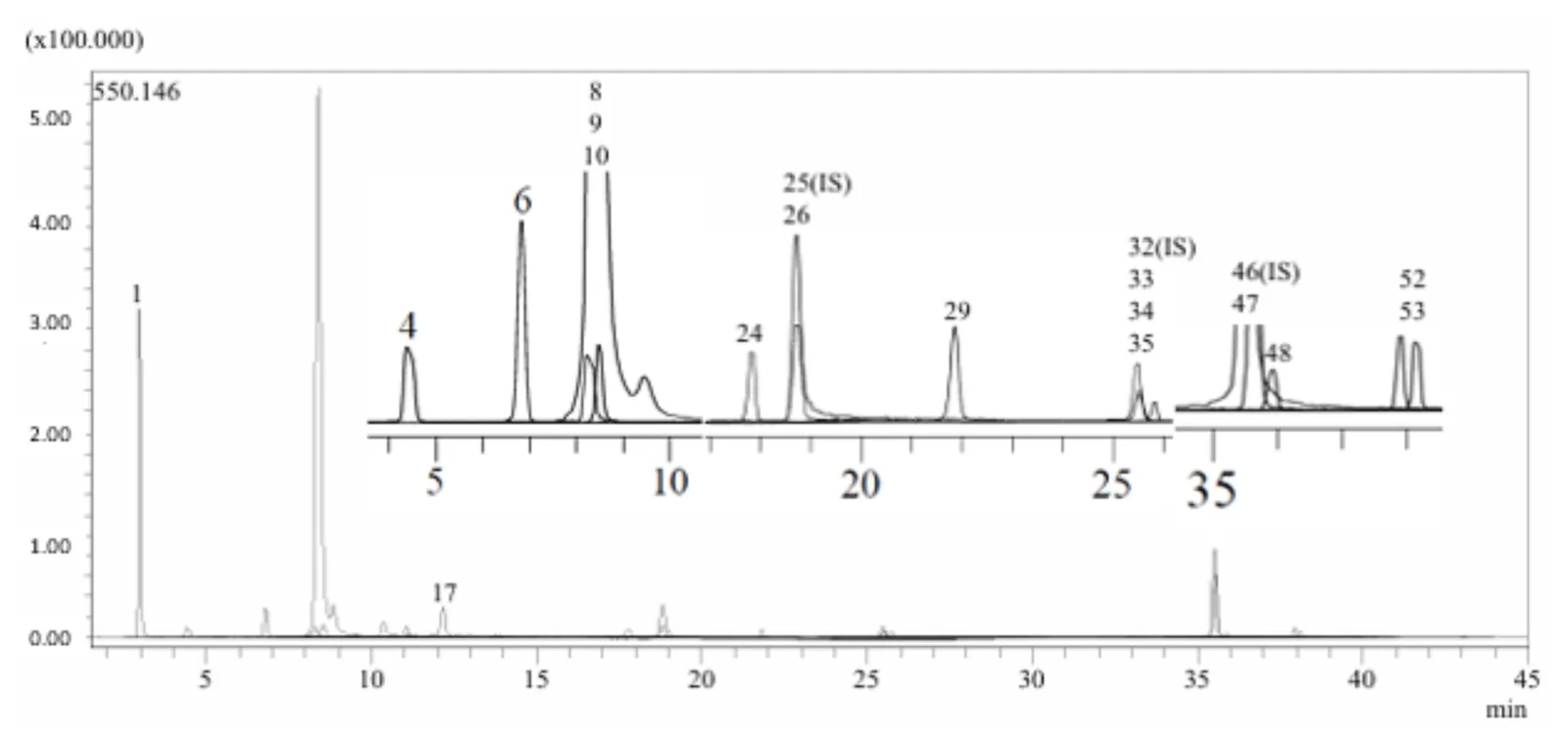
LC–MS/MS chromatogram of the *R. rotundifolia* extract. 1. Quinic acid, 4. Gallic acid, 6. Protocatechuic acid, 8. Gentisic acid, 9. Chlorogenic acid, 10. Protocatechuic aldehyde, 17. Caffeic acid, 24. p-Coumaric acid, 25. Ferulic acid-D3-IS, 26. Ferulic acid, 29. Salicylic acid, 32. Rutin-D3-IS, 33. Rutin, 34. Isoquercitrin, 35. Hesperidin, 46. Quercetin-D3-IS, 47. Quercetin, 48. Naringenin, 52. Kaempferol, 53. Apigenin.

Because *R. rotundifolia* extract exhibited higher inhibitory activity against all three enzymes compared with the other extracts, its chemical profile was further investigated. A previously validated analytical method was employed for the quantitative chemical analysis [27]. The chromatogram of the standard compounds is presented in Figure 1. Liquid chromatography–tandem mass spectrometry (LC–MS/MS) analysis revealed that quinic acid was the most abundant compound (20.79 mg/g extract) in the extract prepared from the aerial parts of *R. rotundifolia*. The identified compounds in the *R. rotundifolia* extract and their corresponding quantities are presented in Table 5. The chromatogram of the extract is presented in Figure 2. The validation parameters for the standard compounds are provided in the Supplementary Table.

## 4. Discussion

Plants may exert beneficial effects in the prevention of obesity and diabetes, which are among the most prevalent chronic metabolic diseases worldwide. Clinical studies have reported that plant metabolites may serve as safe and cost-effective agents for the management of obesity and diabetes [28]. Furthermore, plant-derived compounds may enhance antidiabetic activity through their antioxidant properties and could serve as potential complementary therapeutic agents, particularly in type 2 diabetes [29].

To the best of our knowledge, this is the first study to evaluate the antiobesity and antidiabetic effects of the aerial parts of *R. davisiana*, *R. peregrina*, and *R. rotundifolia*. The aim of this study was to evaluate the effects of three *Rubia* species on carbohydrate and lipid metabolism. In this context, the inhibitory activities of the extracts against α-glucosidase, pancreatic lipase, and α-amylase were evaluated. In addition, the antioxidant activities of the extracts was assessed using five different methods.

The choice of solvent in the extraction process is a critical factor influencing the phytochemical composition and biological activity of the extract. Hydroalcoholic solvents facilitate the extraction of phytochemicals with a broad range of polarities into the solution [30]. In this context, 80% ethanol represents a suitable solvent for extracting biologically active constituents. Due to its moderate polarity, 80% ethanol enables the efficient extraction of diverse phytochemical compounds [31,32]. Based on these considerations, 80% ethanol was selected as the extraction solvent.

The aerial parts and roots of plants belonging to the genus *Rubia* have been used in traditional medicine [33]. In a study comparing the aerial parts and roots of *R. tinctorum*, the aerial part extract exhibited higher DPPH radical scavenging activity and greater ferric reducing power [34]. The aerial parts of *R. cordifolia* have been reported to exhibit nitric oxide inhibitory and antiviral activities [35]. In this context, research focusing on extracts obtained from the aerial parts of *Rubia* species remains limited [36]. Based on these findings, the biological activities of the aerial parts of selected *Rubia* species were investigated.

In the present study, *R. rotundifolia* extract exhibited high inhibitory activity against α-glucosidase, which is an enzyme involved in carbohydrate metabolism. At 2 mg/mL, *R. rotundifolia* extract exhibited higher DPPH and ABTS radical scavenging activities as well as greater metal chelating activity compared with the other extracts. When ferric reducing power was evaluated, *R. davisiana* extract (2 mg/mL) exhibited the highest absorbance value.

To the best of our knowledge, the chemical profile of *R. rotundifolia* extract has not previously been investigated using LC–MS/MS analysis. Quinic acid and chlorogenic acid were identified as the most abundant compounds in the aerial parts of *R. rotundifolia*. Several phytochemical studies on *Rubia* species have been reported in the literature. Wang et al. [37] identified the bioactive naphthohydroquinone derivative mollugin in the roots of *R. cordifolia*. A total of 28 anthraquinones, including purpurin, alizarin, rubiadin, munjistin, techoquinone, and xanthopurpurin, have been isolated from this species [38]. Cai et al. [39] detected gallic acid in the methanolic extract of *R. cordifolia* roots. Aboud [40] reported that the roots of *R. tinctorum* contain kaempferol. Therefore, the present findings are consistent with those reported by Aboud [40] and Cai et al. [39]. Zohra et al. identified hesperidin (3.98 μg/mg) as the major component in the aerial parts of *R. tinctorum*. Additionally, chlorogenic acid, 4-hydroxybenzoic acid, catechin, epicatechin, hesperidin, rutin, caffeic acid, p-coumaric acid, and protocatechuic acid were detected in the extract of *R. tinctorum* [34]. Similarly, chlorogenic acid, hesperidin, rutin, caffeic acid, p-coumaric acid, and protocatechuic acid were detected in the present study. However, catechin, epicatechin, and 4-hydroxybenzoic acid were not detected. The variations in chemical composition observed among *Rubia* species across different studies may be attributed to differences in growing conditions, such as climate, temperature, and altitude.

In a study investigating the antiproliferative and antioxidant effects of methanol extracts prepared from the roots, stems, and leaves of *R. cordifolia*, the root extracts exhibited both the highest antioxidant activity and the highest cytotoxicity [41]. Joharapurkar et al. [42] reported that an alcoholic extract prepared from *R. cordifolia* roots exerted antioxidant effects in an ethanol-induced immunosuppression model. In a study evaluating the DPPH radical scavenging activities of methanol, hexane, chloroform, ethyl acetate, and n-butanol fractions obtained from *R. cordifolia* roots, the hexane and ethyl acetate fractions exhibited the highest activities at 1 mg/mL (96.74% and 94.76%, respectively) [43]. Zohra et al. [34] investigated the antioxidant activities of 80% methanol extracts prepared from the roots and aerial parts of *R. tinctorum*. The extract prepared from the aerial parts exhibited higher DPPH radical scavenging activity (83.23%) and greater ferric reducing power (absorbance value: 1.51). Maxia et al. [44] reported that the extract of the aerial parts of *R. peregrina* exhibited notable DPPH radical scavenging activity (IC_50_: 55.6 μg/mL). Consistent with the findings of Maxia et al., *R. peregrina* extract (2 mg/mL) exhibited high DPPH radical scavenging activity. Furthermore, *R. rotundifolia* and *R. davisiana* extracts also exhibited high DPPH radical scavenging activity. Özgen et al. [45] reported that the ethyl acetate fraction of *R. peregrina* roots exhibited 96% antioxidant activity at a concentration of 0.25% in the thiobarbituric acid assay. In the present study, *R. peregrina* extract (0.5 mg/mL) exhibited low metal chelating activity, low ABTS radical scavenging activity, and limited ferric reducing power. Therefore, the present findings differ from those reported by Özgen et al. [45] in this regard. This discrepancy may be attributed to differences in the extraction procedures and analytical methods employed.

The ethyl acetate fraction prepared from the roots of *Rubia yunnanensis* significantly reduced triglyceride accumulation in HepG2 cells. The root extract significantly prevented the increase in triglyceride levels in mice in a dose-dependent manner [46]. The antiobesity effects of ethanol and aqueous extracts of *R. cordifolia* leaves were investigated in a cafeteria diet-induced obesity model in rats, and the extracts reduced feed intake, basal metabolic rate, weight gain, and the obesity index [47]. In another study, an 80% methanolic extract (0.2 mg/mL) of *R. akane* inhibited pancreatic lipase activity by 58.7% [48]. In the present study, *R. rotundifolia* ethanol extract (2 mg/mL) exhibited low pancreatic lipase inhibitory activity, and no significant antiobesity effect was observed. This discrepancy from previous studies may be attributed to differences in experimental methods and the *Rubia* species investigated. The in vitro pancreatic lipase inhibitory activities of *R. rotundifolia* and *R. peregrina* extracts were significantly lower than those of orlistat, which is used in the treatment of obesity. Therefore, the low pancreatic lipase inhibitory activity demonstrated by both extracts may not be clinically meaningful. In this context, further studies are warranted to investigate the potential antiobesity effects of the extracts through mechanisms other than pancreatic lipase inhibition.

Rani et al. [49] reported that a methanol extract (98.33 mg/mL) of *R. cordifolia* roots inhibited α-amylase activity by 50%. In the present study, the α-amylase inhibitory activities of the extracts were lower than those reported by Rani et al. [49]. This discrepancy may be attributed to differences in the plant species investigated. The α-amylase inhibitory activities of the three extracts (*R. davisiana, R. peregrina*, and *R. rotundifolia)* were significantly lower than that of acarbose. The methanol extract of *R. cordifolia* leaves significantly reduced blood glucose levels in mice with alloxan-induced diabetes [50]. A previous study revealed that the chloroform extract of *R. cordifolia* exhibited α-glucosidase inhibitory activity [51]. A methanol extract prepared from *R. cordifolia* roots exhibited α-glucosidase inhibitory activity (IC_50_: 95.34 μg/mL). Researchers reported that *R. cordifolia* extract may represent a potential antidiabetic agent for the management of hyperglycemia due to its antidiabetic and antioxidant activities [49]. The chloroform extract of *Rubia manjith* roots exhibited high α-glucosidase inhibitory activity (72.9%, IC_50_: 32.9 μg/mL) [52]. In another study, anthraquinone derivatives isolated from *Rubia philippinensis* inhibited α-glucosidase in a concentration-dependent manner [53]. Consistent with previous studies, the extract prepared from the aerial parts of *R. rotundifolia* exhibited α-glucosidase inhibitory activity. This α-glucosidase inhibitory activity may be associated with the abundant presence of chlorogenic acid and quinic acid. Previous studies have shown that quinic acid and chlorogenic acid exhibit high α-glucosidase inhibitory activity [54–56]. The α-glucosidase inhibitory activity of *Pistacia atlantica* gall extracts has been reported to be associated with quinic acid [57]. Similarly, the chlorogenic acid-rich fraction of *Smilax aristolochiifolia* root extract was reported to be a potent α-glucosidase inhibitor [58]. Another study demonstrated that chlorogenic acid inhibited α-glucosidase in a dose-dependent manner and suppressed the increase in blood glucose levels in diabetic rats [59]. In a previous study, quinic acid and chlorogenic acid were reported to bind to the α-glucosidase structure, forming a stable complex [60]. In this context, recent studies suggest that quinic acid and chlorogenic acid are promising compounds with antidiabetic potential [61–63].

To the best of our knowledge, this is the first study investigating the antidiabetic and antiobesity activities of *R. davisiana*, *R. peregrina*, and *R. rotundifolia*. Although *R. rotundifolia* extract exhibited α-amylase inhibitory activity at all investigated concentrations, this activity was lower than that of acarbose. In contrast, *R. rotundifolia* extract exhibited moderate α-glucosidase inhibitory activity. All three extracts showed no significant pancreatic lipase inhibitory activity. LC–MS/MS analysis revealed that the aerial parts of *R. rotundifolia* are rich in quinic acid and chlorogenic acid. In this context, further comprehensive in vitro and in vivo studies are warranted to elucidate the antidiabetic potential of *R. rotundifolia* extracts.

## Supplementary materials

**Supplementary Table:** Validation parameters for the standard compounds.

No	Analytes	RT ^a^	M.I. (m/z) ^b^	F.I. (m/z) ^c^	Ion. mode	Equation	*r* *^2^* d	*RSD*% ^e^		Linearity Range (mg/L)	*LOD*/ *LOQ* (μg/L) ^f^	Recovery (%)		*U* g	Gr. No ^i^
								Interday	Intraday			Interday	Intraday		
1	Quinic acid	3.0	190.8	93.0	Neg	*y*=−0.0129989+2.97989×	0.996	0.69	0.51	0.1–5	25.7/33.3	1.0011	1.0083	0.0372	1
2	Fumaric aid	3.9	115.2	40.9	Neg	*y*=−0.0817862+1.03467×	0.995	1.05	1.02	1–50	135.7/167.9	0.9963	1.0016	0.0091	1
3	Aconitic acid	4.0	172.8	129.0	Neg	*y*=−0.7014530+32.9994×	0.971	2.07	0.93	0.1–5	16.4/31.4	0.9968	1.0068	0.0247	1
4	Gallic acid	4.4	168.8	79.0	Neg	*y*=0.0547697+20.8152×	0.999	1.60	0.81	0.1–5	13.2/17.0	1.0010	0.9947	0.0112	1
5	Epigallocatechin	6.7	304.8	219.0	Neg	*y*=−0.00494986+0.0483704×	0.998	1.22	0.73	1–50	237.5/265.9	0.9969	1.0040	0.0184	3
6	Protocatechuic acid	6.8	152.8	108.0	Neg	*y*=0.211373+12.8622×	0.957	1.43	0.76	0.1–5	21.9/38.6	0.9972	1.0055	0.0350	1
7	Catechin	7.4	288.8	203.1	Neg	*y*=−0.00370053+0.431369×	0.999	2.14	1.08	0.2–10	55.0/78.0	1.0024	1.0045	0.0221	3
8	Gentisic acid	8.3	152.8	109.0	Neg	*y*=−0.0238983+12.1494×	0.997	1.81	1.22	0.1–5	18.5/28.2	0.9963	1.0077	0.0167	1
9	Chlorogenic acid	8.4	353.0	85.0	Neg	*y*=0.289983+36.3926×	0.995	2.15	1.52	0.1–5	13.1/17.6	1.0000	1.0023	0.0213	1
10	Protocatechuic aldehyde	8.5	137.2	92.0	Neg	*y*=0.257085+25.4657×	0.996	2.08	0.57	0.1–5	15.4/22.2	1.0002	0.9988	0.0396	1
11	Tannic acid	9.2	182.8	78.0	Neg	*y*=0.0126307+26.9263×	0.999	2.40	1.16	0.05–2.5	15.3/22.7	0.9970	0.9950	0.0190	1
12	Epigallocatechin gallate	9.4	457.0	305.1	Neg	*y*=−0.0380744+1.61233×	0.999	1.30	0.63	0.2–10	61.0/86.0	0.9981	1.0079	0.0147	3
13	1,5-dicaffeoylquinic acid	9.8	515.0	191.0	Neg	*y*=−0.0164044+16.6535×	0.999	2.42	1.48	0.1–5	5.8/9.4	0.9983	0.9997	0.0306	1
14	4-hydroxybenzoic acid	10.5	137.2	65.0	Neg	*y*=−0.0240747+5.06492×	0.999	1.24	0.97	0.2–10	68.4/88.1	1.0032	1.0068	0.0237	1
15	Epicatechin	11.6	289.0	203.0	Neg	*y*=−0.0172078+0.0833424×	0.996	1.47	0.62	1–50	139.6/161.6	1.0013	1.0012	0.0221	3
16	Vanillic acid	11.8	166.8	108.0	Neg	*y*=−0.0480183+0.779564×	0.999	1.92	0.76	1–50	141.9/164.9	1.0022	0.9998	0.0145	1
17	Caffeic acid	12.1	179.0	134.0	Neg	*y*=0.120319+95.4610×	0.999	1.11	1.25	0.05–2.5	7.7/9.5	1.0015	1.0042	0.0152	1
18	Syringic acid	12.6	196.8	166.9	Neg	*y*=−0.0458599+0.663948×	0.998	1.18	1.09	1–50	82.3/104.5	1.0006	1.0072	0.0129	1
19	Vanillin	13.9	153.1	125.0	Poz	*y*=0.00185898+20.7382×	0.996	1.10	0.85	0.1–5	24.5/30.4	1.0009	0.9967	0.0122	1
20	Syringic aldehyde	14.6	181.0	151.1	Neg	*y*=−0.0128684+7.90153×	0.999	2.51	0.77	0.4–20	19.7/28.0	1.0001	0.9964	0.0215	1
21	Daidzin	15.2	417.1	199.0	Poz	*y*=9.45747+152.338×	0.996	2.25	1.32	0.05–2.5	7.0/9.5	0.9955	1.0017	0.0202	2
22	Epicatechin gallate	15.5	441.0	289.0	Neg	*y*=−0.0142216+1.06768×	0.997	1.63	1.28	0.1–5	19.5/28.5	0.9984	0.9946	0.0229	3
23	Piceid	17.2	391.0	135/106.9	Poz	*y*=0.00772525+25.4181×	0.999	1.94	1.16	0.05–2.5	13.8/17.8	1.0042	0.9979	0.0199	1
24	*p*-Coumaric acid	17.8	163.0	93.0	Neg	*y*=0.0249034+18.5180×	0.999	1.92	1.43	0.1–5	25.9/34.9	1.0049	1.0001	0.0194	1
25	Ferulic acid-D3-IS	18.8	196.2	152.1	Neg	N.A.	N.A.	N.A.	N.A.	N.A.	N.A.	N.A.	N.A.	0.0170	1
26	Ferulic acid	18.8	192.8	149.0	Neg	*y*=−0.0735254+1.34476×	0.999	1.44	0.53	1–50	11.8/15.6	0.9951	0.9976	0.0181	1
27	Sinapic acid	18.9	222.8	193.0	Neg	*y*=−0.0929932+0.836324×	0.999	1.45	0.52	0.2–10	65.2/82.3	1.0031	1.0037	0.0317	1
28	Coumarin	20.9	146.9	103.1	Poz	*y*=0.0633397+136.508×	0.999	2.11	1.54	0.05–2.5	214.2/247.3	0.9950	0.9958	0.0383	1
29	Salicylic acid	21.8	137.2	65.0	Neg	*y*=0.239287+153.659×	0.999	1.48	1.18	0.05–2.5	6.0/8.3	0.9950	0.9998	0.0158	1
30	Cyanoside	23.7	447.0	284.0	Neg	*y*=0.280246+6.13360×	0.997	1.56	1.12	0.05–2.5	12.1/16.0	1.0072	1.0002	0.0366	2
31	Miquelianin	24.1	477.0	150.9	Neg	*y*=−0.00991585+5.50334×	0.999	1.31	0.95	0.1–5	10.6/14.7	0.9934	0.9965	0.0220	2
32	Rutin-D3-IS ^h^	25.5	612.2	304.1	Neg	N.A.	N.A.	N.A.	N.A.	N.A.	N.A.	N.A.	N.A.	N.A.	2
33	Rutin	25.6	608.9	301.0	Neg	*y*=−0.0771907+2.89868×	0.999	1.38	1.09	0.1–5	15.7/22.7	0.9977	1.0033	0.0247	2
34	Isoquercitrin	25.6	463.0	271.0	Neg	*y*=−0.111120+4.10546×	0.998	2.13	0.78	0.1–5	8.7/13.5	1.0057	0.9963	0.0220	2
35	Hesperidin	25.8	611.2	449.0	Poz	*y*=0.139055+13.2785×	0.999	1.84	1.35	0.1–5	19.0/26.0	0.9967	1.0043	0.0335	2
36	*o*-Coumaric acid	26.1	162.8	93.0	Neg	*y*=0.00837193+11.2147×	0.999	2.11	1.46	0.1–5	31.8/40.4	1.0044	0.9986	0.0147	1
37	Genistin	26.3	431.0	239.0	Neg	*y*=1.65808+7.57459×	0.991	2.01	1.28	0.1–5	14.9/21.7	1.0062	1.0047	0.0083	2
38	Rosmarinic acid	26.6	359.0	197.0	Neg	*y*=−0.0117238+8.04377×	0.999	1.24	0.86	0.1–5	16.2/21.2	1.0056	1.0002	0.0130	1
39	Ellagic acid	27.6	301.0	284.0	Neg	*y*=0.00877034+0.663741×	0.999	1.57	1.23	0.4–20	56.9/71.0	1.0005	1.0048	0.0364	1
40	Cosmosiin	28.2	431.0	269.0	Neg	*y*=−0.708662+8.62498×	0.998	1.65	1.30	0.1–5	6.3/9.2	0.9940	0.9973	0.0083	2
41	Quercitrin	29.8	447.0	301.0	Neg	*y*=−0.00153274+3.20368×	0.999	2.24	1.16	0.1–5	4.8/6.4	0.9960	0.9978	0.0268	2
42	Astragalin	30.4	447.0	255.0	Neg	*y*=0.00825333+3.51189×	0.999	2.08	1.72	0.1–5	6.6/8.2	0.9968	0.9957	0.0114	2
43	Nicotiflorin	30.6	592.9	255.0/284.0	Neg	*y*=0.00499333+2.62351×	0.999	1.48	1.23	0.05–2.5	11.9/16.7	0.9954	1.0044	0.0108	2
44	Fisetin	30.6	285.0	163.0	Neg	*y*=0.0365705+8.09472×	0.999	1.75	1.19	0.1–5	10.1/12.7	0.9980	1.0042	0.0231	3
45	Daidzein	34.0	253.0	223.0	Neg	*y*=−0.0329252+6.23004×	0.999	2.18	1.73	0.1–5	9.8/11.6	0.9926	0.9963	0.0370	3
46	Quercetin-D3-IS ^h^	35.6	304.0	275.9	Neg	N.A.	N.A.	N.A.	N.A.	N.A.	N.A.	N.A.	N.A.	N.A.	3
47	Quercetin	35.7	301.0	272.9	Neg	*y*=+0.00597342+3.39417×	0.999	1.89	1.38	0.1–5	15.5/19.0	0.9967	0.9971	0.0175	3
48	Naringenin	35.9	270.9	119.0	Neg	*y*=−0.00393403+14.6424×	0.999	2.34	1.69	0.1–5	2.6/3.9	1.0062	1.0020	0.0392	3
49	Hesperetin	36.7	301.0	136.0/286.0	Neg	*y*=+0.0442350+6.07160×	0.999	2.47	2.13	0.1–5	7.1/9.1	0.9998	0.9963	0.0321	3
50	Luteolin	36.7	284.8	151.0/175.0	Neg	*y*=−0.0541723+30.7422×	0.999	1.67	1.28	0.05–2.5	2.6/4.1	0.9952	1.0029	0.0313	3
51	Genistein	36.9	269.0	135.0	Neg	*y*=−0.00507501+12.1933×	0.999	1.48	1.19	0.05–2.5	3.7/5.3	1.0069	1.0012	0.0337	3
52	Kaempferol	37.9	285.0	239.0	Neg	*y*=−0.00459557+3.13754×	0.999	1.49	1.26	0.05–2.5	10.2/15.4	0.9992	0.9990	0.0212	3
53	Apigenin	38.2	268.8	151.0/149.0	Neg	*y*=0.119018+34.8730×	0.998	1.17	0.96	0.05–2.5	1.3/2.0	0.9985	1.0003	0.0178	3
54	Amentoflavone	39.7	537.0	417.0	Neg	*y*=0.727280+33.3658×	0.992	1.35	1.12	0.05–2.5	2.8/5.1	0.9991	1.0044	0.0340	3
55	Chrysin	40.5	252.8	145.0/119.0	Neg	*y*=−0.0777300+18.8873×	0.999	1.46	1.21	0.05–2.5	1.5/2.8	0.9922	1.0050	0.0323	3
56	Acacetin	40.7	283.0	239.0	Neg	*y*=−0.559818+163.062×	0.997	1.67	1.28	0.02–1	1.5/2.5	0.9949	1.0011	0.0363	3

a R.T.: Retention time,

b MI (*m/z):* Molecular ions of the standard analytes (m/z ratio),

c FI (*m/z):* Fragment ions

d *r*^2^: Coefficient of determination,

e *RSD*: Relative standard deviation,

f *LOD*/*LOQ* (μg/L): Limit of detection/quantification,

g *U* (%): percent relative uncertainty at 95% confidence level (*k* = 2),

h IS: Internal standard,

i Gr. No: Represents grouping of internal standards, these numbers indicate which IS stands for which phenolic compound.

## Figures and Tables

**Table 1:** Used plant parts, collection locations, and herbarium voucher numbers of the investigated *Rubia* species.

Plant name	Used parts	Herbarium no	Collection sites, dates
*R. davisiana*	Aerial parts	GUL 118/10/1-1	Antalya, Avlan Lake surroundings, between Elmalı and Finike, under *Quercus* sp., 30 July 2024
*R. peregrina*	Aerial parts	GUL 118/10/4-1	Çanakkale, between Ayvacık and Çanakkale, roadsides, 9 June 2024
*R. rotundifolia*	Aerial parts	GUL 118/10/2-1	Hatay, Dörtyol, near Topaktaş, *Fagus* sp. Forest, 7 May 2024

**Table 2:** Extraction yield, total antioxidant capacity, total phenolic content, and total flavonoid content of the extracts.

Extract	Yield % (w/w)	Total antioxidant capacity^a^ (Mean ± SD)	Total phenolic content^b^ (Mean ± SD)	Total flavonoid content^c^ (Mean ± SD)
*R. davisiana*	21.60	117.37 ± 7.83	66.77 ± 1.24	59.44 ± 1.52
*R. peregrina*	18.47	102.29 ± 8.48	52.15 ± 3.00	48.08 ± 2.16
*R. rotundifolia*	12.94	147.35 ± 7.54	60.55 ± 1.38	54.90 ± 1.62

a mg ascorbic acid equivalents (AAE)/g extract;

b mg gallic acid equivalents (GAE)/g extract;

c mg quercetin equivalents (QE)/g extract.

**Table 3:** Antioxidant activity assay results of the extracts.

	(mg/mL)	Antioxidant activity assays			
		DPPH radical scavenging activity inhibition% ± SD	ABTS radical scavenging activity inhibition% ± SD	Metal chelating capacity % ± SD	Ferric reducing power absorbance ± SD
*R. davisiana*	0.5	83.65 ± 0.55***	26.07 ± 1.15***	-	0.572 ± 0.01***
	1	85.17 ± 0.42***	26.68 ± 0.91***	-	1.047 ± 0.07***
	2	82.18 ± 1.14***	33.27 ± 0.93***	19.07 ± 2.74**	1.820 ± 0.13***
*R. peregrina*	0.5	29.18 ± 0.89**	8.28 ± 0.76^ns^	-	0.195 ± 0.02***
	1	79.81 ± 1.67***	12.86 ± 2.93*	-	0.474 ± 0.01***
	2	82.18 ± 0.88***	18.59 ± 2.57***	9.46 ± 1.10*	0.840 ± 0.12***
*R. rotundifolia*	0.5	53.55 ± 1.23***	4.65 ± 0.57^ns^	-	0.356 ± 0.03***
	1	78.39 ± 2.45***	17.37 ± 2.86***	-	0.598 ± 0.08***
	2	83.91 ± 0.42***	33.94 ± 0.93***	21.16 ± 1.23***	1.140 ± 0.10***
References	0.5	88.70 ± 0.55^a^***	99.26 ± 0.62^b^***	99.95 ± 0.25^c^***	3.697 ± 0.08^d^***
	1	89.75 ± 0.16^a^***	98.72 ± 0.12^b^***	99.84 ± 0.66^c^***	3.735 ± 0.13^d^***
	2	90.17 ± 0.14^a^***	98.25 ± 0.71^b^***	99.95 ± 0.09^c^***	3.856 ± 0.03^d^***

a Ascorbic acid;

b Gallic acid;

c EDTA;

d Quercetin;

* p < 0.05;

** p < 0.01;

*** p < 0.001; –: no effect; ns: no statistically significant difference;

SD: standard deviation. The data were compared with the negative control used in the tests. Reference compounds and extracts were evaluated at the same concentrations under identical analytical conditions.

**Table 4:** Enzyme inhibition assay results of the extracts.

	(mg/mL)	Enzyme inhibition % ± SD		
		α-glucosidase	α-amylase	Pancreatic lipase
*R. davisiana*	0.5	-	-	-
	1	-	2.33 ± 0.54^ns^	-
	2	-	13.26 ± 2.81*	-
*R. peregrina*	0.5	-	-	-
	1	-	3.26 ± 1.43^ns^	-
	2	35.95 ± 1.77**	14.95 ± 0.91**	12.65 ± 2.91*
*R. rotundifolia*	0.5	40.37 ± 1.79***	13.26 ± 1.32*	-
	1	47.66 ± 1.48***	14.82 ± 2.16**	-
	2	74.70 ± 0.63***	18.88 ± 2.48**	13.18 ± 0.64*
References	0.5	99.60 ± 0.23^a^***	88.92 ± 1.34^a^***	53.85 ± 0.54^b^***
	1	99.80 ± 0.38^a^***	91.92 ± 0.64^a^***	68.46 ± 2.72^b^***
	2	99.95 ± 0.38^a^***	93.20 ± 1.28^a^***	63.85 ± 1.63^b^***

a Acarbose;

b Orlistat;

* p < 0.05;

** p < 0.01;

*** p < 0.001;

–: no effect; ns: no statistically significant difference;

SD: standard deviation. The data were compared with the negative control used in the tests. Reference compounds and extracts were evaluated at the same concentrations under identical analytical conditions.

**Table 5:** Chemical composition of *R. rotundifolia* extract determined by LC–MS/MS analysis.

No	Compounds	Extract (mg/g)	No	Compounds	Extract (mg/g)
1	Quinic acid	20.79	29	Salicylic acid	0.051
2	Fumaric acid	N.D.	30	Cyanoside	N.D.
3	Aconitic acid	N.D.	31	Miquelianin	N.D.
4	Gallic acid	0.184	32	Rutin-D3-IS	N.A.
5	Epigallocatechin	N.D.	33	Rutin	0.082
6	Protocatechuic acid	0.606	34	Isoquercitrin	0.084
7	Catechin	N.D.	35	Hesperidin	0.047
8	Gentisic acid	0.12	36	o-Coumaric acid	N.D.
9	Chlorogenic acid	9.928	37	Genistin	N.D.
10	Protocatechuic aldehyde	0.164	38	Rosmarinic acid	N.D.
11	Tannic acid	N.D.	39	Ellagic acid	N.D.
12	Epigallocatechin gallate	N.D.	40	Cosmosiin	N.D.
13	Cynarin	N.D.	41	Quercitrin	N.D.
14	4-hydroxybenzoic acid	N.D.	42	Astragalin	N.D.
15	Epicatechin	N.D.	43	Nicotiflorin	N.D.
16	Vanillic acid	N.D.	44	Fisetin	N.D.
17	Caffeic acid	0.151	45	Daidzein	N.D.
18	Syringic acid	N.D.	46	Quercetin-D3-IS	N.A.
19	Vanillin	N.D.	47	Quercetin	0.441
20	Syringic aldehyde	N.D.	48	Naringenin	0.004
21	Daidzin	N.D.	49	Hesperetin	N.D.
22	Epicatechin gallate	N.D.	50	Luteolin	N.D.
23	Piceid	N.D.	51	Genistein	N.D.
24	p-Coumaric acid	0.069	52	Kaempferol	0.008
25	Ferulic acid-D3-IS	N.A.	53	Apigenin	0.005
26	Ferulic acid	0.126	54	Amentoflavone	N.D.
27	Sinapic acid	N.D.	55	Chrysin	N.D.
28	Coumarin	N.D.	56	Acacetin	N.D.

N.A.: not applicable; N.D.: not detected.
